# Abnormal uterine bleeding and chronic iron deficiency: Number 11 – December 2022

**DOI:** 10.1055/s-0042-1760235

**Published:** 2022-12-29

**Authors:** Venina Viana de Barros, Eliane Azeka Hase, Cristiano Caetano Salazar, Ana Maria Kondo Igai, Fernanda Andrade Orsi, Paulo Francisco Ramos Margarido

**Affiliations:** 1 Departamento de Obstetrícia e Ginecologia, Hospital das Clínicas, Faculdade de Medicina, Universidade de São Paulo, São Paulo, SP, Brazil; 2 Departamento de Obstetrícia e Ginecologia, Hospital das Clínicas, Faculdade de Medicina, Universidade de São Paulo, São Paulo, SP, Brazil; 3 Hospital Moinhos de Vento, Porto Alegre, RS, Brazil; 4 Departamento de Obstetrícia e Ginecologia, Hospital das Clínicas, Faculdade de Medicina, Universidade de São Paulo, São Paulo, SP, Brazil; 5 Departamento de Hematologia, Hospital das Clínicas, Faculdade de Medicina, Universidade de São Paulo, São Paulo, SP, Brazil; Departamento de Hematologia da Universidade Estadual de Campinas, Campinas, SP, Brazil; 6 Departamento de Obstetrícia e Ginecologia, Hospital das Clínicas, Faculdade de Medicina, Universidade de São Paulo, São Paulo, SP, Brazil

## Key points

- Abnormal uterine bleeding (AUB) in menacme is the leading cause of iron deficiency anemia (IDA) and iron deficiency (ID).
- All patients with AUB should be investigated and treated.
- Anemia is one of the most common problems in clinical practice that affects millions of people worldwide.
- Non-pregnant women account for 30% of all anemia cases in the world, and approximately 60% of them have ID.
- Oral iron replacement is the most widespread, especially in cases of milder IDA and ID.
- Intravenous (IV) formulations have gained more space in prescriptions as their safety and efficacy have become more evident.

## Recommendations

- Abnormal uterine bleeding is a very frequent complaint that negatively affects the quality of life since menacme. Investigation for IDA and ID is mandatory in these patients.
- The approach to patients with AUB prioritizes stabilization in acute cases, using mainly hormones and antifibrinolytics to stop bleeding.
- Etiological investigation will guide the therapy in non-acute cases.
- Treatment selection for ID is driven by several factors, including the presence of inflammation, the time available for iron replacement and the anticipated risk of side effects or intolerance.
- The treatment of choice for ID is preferably via oral (VO). The increase in hepcidin by oral iron supplements limits oral absorption when large amounts of iron need to be administered or in the presence of inflammatory conditions.
- Intravenous iron preparations are indicated for the treatment of ID when oral medications are ineffective or cannot be used. They have applicability in a wide range of clinical settings, including chronic inflammatory conditions, perioperative situations, and disorders associated with chronic blood loss.
- Serious adverse events that occur with IV iron are very rare and well-studied, which provides a basis for educating and preparing staff and patients on how iron infusions can be safely and effectively administered.

## Background

One of the most common gynecological complaints worldwide is the occurrence of abnormal uterine bleeding (AUB), a term that refers to abnormalities in the amount, duration or frequency of bleeding from the uterus. With a prevalence of 10-30% among women of reproductive age, it can negatively affect the quality of life and is associated with financial losses, reduced productivity, inadequate health status and greater use of health services. 1 2

## What are the main causes of AUB and how to classify them?

Abnormal uterine bleeding is a symptom, not a diagnosis, and describes bleeding that deviates from the general menstrual pattern of the population. The terms and parameters currently used are described in chart 1 . Abnormal uterine bleeding can also be characterized as acute (severe enough episode that requires immediate intervention), chronic (occurring in most cycles in the previous six months) and intermenstrual bleeding (occurring between defined cycles and predictable menstruation). 1 3

**Chart 1.** Definitions of normal and abnormal menstrual bleeding

Parameter	Descriptive term	Definition
Frequency (interval between the start of each cycle)	Amenorrhea	No bleeding for 90 days
	Infrequent	>38 days
	Normal	24 to 38 days
	Frequent	<24 days
Regularity (variation in duration between the longest and shortest cycle in 12 months)	Regular	≤7 to 9 days
	Irregular	≥10 days
Duration (duration of bleeding)	Normal	≤8 days
	Prolonged	>8 days
Volume (total blood loss)	Mild	Patient perceives as mild
	Normal	Patient considers normal
	Heavy	Patient considers heavy
Intermenstrual bleeding (bleeding between regular menstrual cycles)	Absent (normal)	No bleeding
	Random	Present, not predictable
	Cyclic	Present, predictable (at the beginning, middle or end of the cycle)
Unscheduled bleeding in gonadal steroid users (estrogen ± progestin)	Normal	Absent
	Abnormal	Present
	(Not applicable)	No steroid use

## Causes

The International Federation of Gynecology and Obstetrics (FIGO) classifies the causes of non-pregnancy-related AUB under the PALM-COEIN acronym, referring to Polyps, Adenomyosis, Leiomyoma, Malignancy and hyperplasia, Coagulopathy, Ovulatory dysfunction, Endometrial disorders, Iatrogenic and Not otherwise classified. In general terms, the first group (“PALM”) refers to structural causes (mostly identifiable by imaging exams or histopathology), and the other group (“COEIN”) refers to non-structural causes. The term “dysfunctional uterine bleeding” (DUB), in turn, refers to causes related to hemostasis (“C”), ovulatory dysfunction (“O”) and endometrial primary disorders (“E”), according to the current FIGO classification system. 3

## Should all patients with AUB be investigated and treated?

Management of patients with AUB includes assessment of hemodynamic instability and anemia, identification of the source of bleeding, and exclusion of pregnancy. Initially, it is important to determine whether it is acute or non-acute bleeding. The etiological diagnosis will guide therapy and treatment success. 4 5 However, in situations of acute and severe bleeding, treatment can be instituted to stop acute bleeding, followed by investigation. Although the uterus is often the source of abnormal bleeding, any part of the female genital tract (vulva, vagina) may have externalized vaginal bleeding, and this differential diagnosis is necessary. Initial physical examination may reveal vulvar or cervical lesions, guiding specific therapy. Anamnesis focused on the bleeding pattern, the use of medications and the association of other characteristics, signs and symptoms can guide the investigation, leading to the most likely etiologies ( Chart 2 ).

**Chart 2.** Differential diagnosis of AUB in non-pregnant women of reproductive age

Bleeding pattern	Associated clinical features	Etiologies to consider
Regular, heavy, or prolonged periods	Enlarged uterus on physical examination, with or without palpable masses	Leiomyomas
	Dysmenorrhea Enlarged and softened uterus on physical examination	Adenomyosis
	Family history of blood dyscrasia Symptoms of hemorrhagic diathesis Anticoagulant therapy	Coagulopathies
	Risk factors for uterine cancer	Endometrial carcinoma, uterine sarcoma
Regular periods with intermenstrual bleeding		Endometrial polyp
	Risk factors for uterine cancer	Endometrial carcinoma, uterine sarcoma
	Recent history of cervical or uterine procedure, or delivery/cesarean section, especially if infected	Chronic endometritis
Irregular bleeding, more or less frequent than normal periods, with variable volume and duration		Ovulatory dysfunction
	Hirsutism, acne, obesity	Polycystic ovary syndrome
	Galactorrhea	Hyperprolactinemia
	Recent weight loss or gain Cold or heat intolerance Family history of thyroid disease	Thyroidopathy
	Risk factors for uterine cancer	Endometrial carcinoma, uterine sarcoma

## How to perform the management and follow-up of AUB?

### Acute uterine bleeding

When there is acute and severe blood loss and the patient is anemic and hypovolemic, hypotensive, tachycardic or with orthostatic hypotension, before determining the etiology, measures to stop bleeding are adopted. The first step is reestablishing hemodynamic stability with the use of crystalloids and eventually, the use of vasopressors and blood components.

### Pharmacological measures

The use of high doses of intravenous estrogen causes rapid endometrial growth, stimulates contraction of the uterine arteries, and promotes platelet aggregation and clotting. Intravenous conjugated estrogen 25 mg every four to six hours for the first 24 hours is suggested, followed by a combination of estrogen and progestin for the following days. 1 4 6 Combined oral contraceptives (COCs), more widely available in our country, can also be used to treat acute AUB. A COC with 35 mcg of ethinyl estradiol (or other combination of pills to achieve this dose) three times a day for seven days is indicated. However, both estrogen treatments (oral or intravenous) should be avoided in patients at high risk of thromboembolism. An alternative is the use of multiple doses of progestins, especially in cases where estrogens are contraindicated. Medroxyprogesterone acetate 20 mg three times a day, norethisterone 5 mg three times a day, or another high-dose progestogen can be used for seven days, followed by one dose a day for three weeks. 1 4 Another suggested option in the literature is the use of a gonadotropin-releasing hormone (GnRH) agonist associated with an aromatase inhibitor or GnRh antagonist. 4 All these hormonal options, after a higher loading dose and a lower maintenance dose for a week or period of a menstrual cycle, in general, can be maintained while etiological investigation is performed. In addition to hormonal alternatives, tranexamic acid can be used to manage acute bleeding; 10 mg/kg of body weight are given intravenously every eight hours (most effective) or 20-25 mg/kg orally every eight hours. The use of antifibrinolytics can reduce bleeding by up to 50%. Caution should also be exercised in patients at high risk of thromboembolism. 1

### Nonpharmacological measures

In some emergencies in which hemodynamic instability persists despite the drug treatment instituted, it is necessary to resort to mechanical or surgical procedures. An alternative is to try to tamponade the uterus by inserting a Foley catheter and fill the balloon with 10-30 mL of saline or distilled water. Sometimes it is necessary to perform a uterine curettage to stop bleeding. If severe bleeding persists, uterine artery embolization or even hysterectomy should be considered, depending on reproductive desire and bleeding severity.

### Non-acute uterine bleeding

For these patients, the objective is to continue the diagnostic investigation and institute management directed at the cause. Pelvic ultrasound is the complementary test that provides more data for the management of AUB cases and has sensitivity of 96% and specificity of 14% for uterine abnormalities. Saline-infused sonography may better reveal intracavitary pathologies (such as polyps and fibroids). Histopathological evaluation (endometrial biopsy) is always indicated in postmenopausal patients, in those aged 45 years or older and those at high risk for endometrial carcinoma. According to risk factors in patients with suspected coagulopathy, the platelet count, plasma fibrinogen, prothrombin time and activated partial thromboplastin time should be evaluated. Sometimes it is necessary to follow the investigation with a von Willebrand factor and platelet aggregation tests, as well as with a hemophilia test – especially in patients with reports of ecchymosis or easy bleeding and in those with a suggestive family history. Patients using anticoagulants (coumarins, heparins, direct oral anticoagulants) should have their therapeutic regimen optimized. When an infectious cause is suspected, tests for gonococcus, chlamydia, and trichomoniasis are performed. If hormonal causes are suspected, it is critical to evaluate prolactin, thyroid tests, gonadotropins, and androgens, as well as other ways to diagnose chronic anovulation or polycystic ovary syndrome. Generally speaking, management is different for structural and non-structural etiologies.

### Structural causes

In causes grouped in the first part (“PALM”) of the PALM-COEIN acronym, the aim of treatment is mostly the structural pathology.

### Non-structural causes

The causes grouped in the second part (“COEIN”) of the PALM-COEIN acronym, the so-called “non-structural”, and some structural causes of AUB have the treatment focus on satisfactory control of bleeding, regardless of etiology.

### Levonorgestrel intrauterine system (LNG-IUS)

Continuously released levonorgestrel (20 mcg/day) from the LNG-IUS is the most effective measure to prevent heavy menstrual bleeding, leading to a 71-95% reduction in blood loss by promoting endometrial atrophy.

### Other isolated systemic progestins

Progestins promote endometrial atrophy and have anti-inflammatory action, although it is not fully understood how they reduce uterine bleeding. They can be indicated for most women, especially those with contraindications to the use of estrogens. Continuous oral progestins are effective in the treatment of AUB, reducing bleeding by up to 87% and promoting amenorrhea in a large percentage of women (10%-15%). 7 Progestogens-only contraceptive pills can be used: norethisterone 0.35 mg, desogestrel 75 mcg, drospirenone 5 mg, or micronized progesterone (200-400 mg/day) in continuous use, or from day 5 to day 26 of the menstrual cycle. Injectable depot medroxyprogesterone acetate (150 mg intramuscularly every three months) can promote amenorrhea in up to 24% of women and is an option for women with increased bleeding. However, there is no conclusive evidence regarding the use of injectable progestin in AUB. Likewise, there are not enough studies to indicate the etonorgestrel implant to manage AUB, even though it promotes amenorrhea in 20% of users. 4

### Estrogen and progesterone combinations

Combined oral contraceptives are able to reduce menstrual bleeding by 35-69%. They are a widely available and effective therapeutic option for most cases of AUB without structural change. Monophasic formulations containing 30 to 35 mcg of ethinylestradiol are the most studied, but the most diverse presentations of COCs are effective. 1 4 A quadriphasic formulation containing dienogest with estradiol valerate (10-30 mcg/day) showed a reduction of menstrual volume thus, is an alternative. 8 Other routes (transdermal patch, vaginal ring) are probably as effective as the oral options, and may be superior when there is an indication to avoid first-pass effect.

### Non-hormonal treatments

Tranexamic acid is the most frequently prescribed antifibrinolytic, associated with a 26-54% reduction in the amount of bleeding. When the patient is bleeding, it is used in doses ranging from 1 to 1.5 g, three to four times a day for about three to five days. It can be used by women with the intention of becoming pregnant, but not by women at higher risk of thromboembolism. Non-steroidal anti-inflammatory drugs (NSAIDs) can be used alone or as an adjuvant therapy to some hormonal treatment, reducing the amount of bleeding by 10% to 52%. The most studied medications are mefenamic acid (500 mg orally three times daily) and naproxen (500 mg orally twice daily) while patient is experiencing bleeding. They can be used by women who are trying to get pregnant, but should be avoided by patients with coagulopathies.

### Surgical treatments

Endometrial ablation is a less invasive alternative to hysterectomy for patients with AUB without structural damage. The aim is to destroy the basal layer of the endometrium through a series of methods (laser, thermal balloon, vaporization, cryoablation, bipolar radiofrequency, microwave), preventing its regeneration. This is an option only for those who no longer wish to get pregnant. The amenorrhea rate is 40-50% in one year, with good results in uteri with hysterometry less than 10 cm. 4 Hysterectomy is an exception treatment for structural AUB reserved for patients with no reproductive desire and unsuccessful drug management. However, it is the most effective definitive treatment and achieves high levels of satisfaction.

### Iron deficiency anemia and iron deficiency: consequence of AUB conditions

In the presence of profuse acute uterine bleeding, acute anemia, hypotension, shock and even death can occur if prompt intervention is not performed. Chronic AUB, in turn, is an important cause of ID, as are parasitic infections, gastrointestinal bleeding, and nutritional deficiencies, which can lead to anemia. 2 9 10 Anemia is defined as a condition in which the concentration of hemoglobin (Hb) in the blood is below normal and can be determined by several factors, with 50% of cases comprising IDA or ID. 11 12 The usual symptoms of IDA include weakness, headache, irritability, restless legs syndrome and varying degrees of fatigue and exercise intolerance or pica (perverted appetite for clay or soil, paper, starch, etc.). 12 13 Patients with low ferritin and without anemia may have the same symptoms. Ice pica may also occur, which is considered quite specific for low ferritin. Some patients with low ferritin, with or without anemia, may complain of tongue pain, decreased salivary flow with dry mouth, and atrophy of the lingual papillae. 13 Other patients may present with alopecia, dry skin, devitalized hair, and koilonychia. 14 However, many patients are asymptomatic, with no typical symptoms, and only recognize symptoms retrospectively after treatment. The differential diagnosis of IDA includes parasitic diseases such as malaria, hookworm and schistosomiasis, nutritional causes such as lack of folic acid, vitamin A and vitamin B12, and genetic causes such as hereditary thalassemia-type hemoglobinopathies. 15

## How to make the laboratory diagnosis of IDA and ID?

When IDA or ID is suspected, 16 17 a complete blood count (with RBC indices and peripheral smear evaluation) and ferritin levels should be requested ( Chart 3 ). 18

**Chart 3.** Laboratory parameters to define iron deficiency anemia (IDA) and iron deficiency (ID)

	Adult normal values	IDA	Latent ID	IDA refractory to iron treatment	Anemia of chronic disease	IDA + anemia of inflammation
Serum iron (μmol/L)	10-30	↓	N/↓	↓	↓	N/↓
Transferrin saturation (%)	20-45	<20	N/↓	<10	N/↓	<20
Serum ferritin (μg/L)	20-200 (F) 40-300 (M)	<30	<30	Variable	>100	30-300
Reticulocyte hemoglobin (pg)	>29	<29	<29	<29	<29	<29
Hemoglobin (g/dL)	>12 (F) >13 (M)	↓	N	↓	↓	↓
MCV (fL)	80-100	↓	N/↓	↓	N/↓	N/↓

Source: Adapted from Elstrott et al. (2020) 18

Other measurements, such as serum iron, transferrin, and transferrin saturation, are not mandatory. Patients with IDA have low serum iron, high transferrin, and low transferrin saturation. 12 13 Reticulocyte hemoglobin (Ret-Hb) is a good indicator of the amount of available iron, and its dosage does not interfere with inflammatory processes. 19 According to the diagnostic standards of the World Health Organization, IDA is mild to moderate if Hb is between 7 and 12 g/dL, and severe, if Hb is less than 7 g/dL, with small variations according to age, sex or presence of pregnancy. 20 For the adult female population, Hb values below 12 g/dL are considered as anemia and for men, Hb values below 13 g/dL. 20 21 Although Hb is widely used for the evaluation of IDA, it has low specificity and sensitivity, and a biomarker of iron status, such as serum ferritin, should be requested together. 21 Serum ferritin concentration is the most reliable marker of iron storage in the body. Normal values range from 30 to 200 ng/mL (mcg/L), and there is no clinical situation in which low rates do not mean ID. As long as patients with IDA do not have infection or associated inflammatory disease, the cutoff value of 30 ng/mL gives better diagnostic efficiency, with sensitivity of 92% and specificity of 98%. As ferritin is an acute-phase reactor with increased levels in inflammatory, infectious, malignant, or liver diseases, falsely elevated ferritin may be found in the presence of these diseases and IDA. The effect of inflammation on ferritin is to increase it threefold. Therefore, in these patients, the golden rule is to divide the ferritin value by 3, and values less than or equal to 20 ng/mL suggest concomitant IDA. 22

## What are the main treatments for iron deficiency?

The main treatments for IDA and ID are iron replacement, correction of nutritional aspects and treatment of AUB. The goal of iron replacement is to provide enough iron to normalize Hb concentrations and replenish iron storage, thereby improving quality of life and symptoms. 13 Regardless of the presence of symptoms, all patients with IDA and most of those with ID without anemia should be treated. 23 24 There are two distinct approaches: prevention strategies targeting populations at risk, such as patients with AUB, and active iron supplementation approaches in confirmed IDA. 13

## Nutritional guidance

It is recommended to increase the intake of meat, the main source of heme iron; it is estimated that 100 g of meat corresponds to 1 kg of beans (non-heme iron). Concomitant consumption of fruit juice with vitamin C enhances the absorption of iron from the diet, and the use of an iron pan to prepare meals is also part of the guidelines. It is recommended not to mix milk and tea at the same meal and avoid whole grain cereals and chocolate as a dessert during the period of treatment with ferrous salt. These recommendations are not necessary when ferric salts are used in the treatment, because in these compounds, iron is chelated with sugar or amino acids, and there is no interaction of its absorption with food in general. Foods rich in ascorbic acid (cashew, legumes, guava) and meats in general, favor the absorption of non-heme iron, while phytates, phosphates and carbonates (pineapple, vegetables, milk), tannin (tea, coffee), phosphoprotein (yolk eggs) and drugs that raise gastric pH (antacids, proton pump inhibitors, histamine H2 blockers) make absorption of non-heme iron difficult. Although intestinal iron absorption can increase significantly when iron is deficient (from less than 1% to more than 50% of the iron present in the diet), dietary correction alone is not usually sufficient to treat patients with IDA. 13 16

## When and how to prescribe oral iron? What are the main indications and contraindications?

Oral iron replacement is undoubtedly the most widespread, especially for lighter IDA and ID cases. However, doubts about the dosage are common and it is not uncommon to find situations when the drug is apparently ineffective. The recommended therapeutic dose is 2 to 5 mg/kg/day for a period sufficient to normalize Hb values – one to two months – and restore normal body iron stores – two to six months or until serum ferritin is greater than 30-50 ng/mL. 16 Therefore, the duration of treatment varies widely depending on the severity of ID and its cause. In practice, the recommended dose for adult individuals is 150 to 200 mg of elemental iron per day, and the administration of daily doses greater than 200 mg is not recommended, as, in this case, the intestinal mucosa acts as a barrier, preventing the absorption of the metal, and the proportion absorbed decreases significantly. 12 16 Chart 4 shows the products and doses available for oral treatment.

**Chart 4.** Main compounds with iron salts available for the oral treatment of iron deficiency anemia (IDA) and iron deficiency (ID)

COMPOUND	Total amount of iron	Amount of elemental iron	Registration on the Anvisa website*
Ferrous sulfat (mg)	190	60 40	Henfer/Anemifer Furp/Farmanguinhos/Nesh
Ferric glycinate (mg)	150, 300, 500	30, 60, 100	Neutrofer
Ferripolymaltose (mg)	333, 33/357	100	Endofer, Noripurum

Research has shown that high doses of iron VO induce greater production of hepcidin, a protein produced by the liver that controls serum iron by blocking intestinal absorption and the release of iron from stores. 12 Therefore, the use in sequence or in overdose may paradoxically take away the effect of the drug. In some studies, lower doses of elemental iron per day, 15 to 20 mg, have shown equal effectiveness compared to higher doses, likely because of this mechanism. In addition to ensuring adequate absorption, dosage adjustments allow for better control of side effects (diarrhea, constipation, epigastric pain, nausea, dark-colored stools). 18 Numerous oral iron formulations are available and mostly, they are all equally effective, as long as they are taken. 23 Iron absorption from intestinal mucosal cells occurs through divalent metal transporter 1 (DMT1), a protein located in the duodenum and upper jejunum. Once in the cell, ferroportin transports iron through the cell into the blood, where it is bound by transferrin. The paradigm for iron replacement evolved as evidence began to emerge suggesting that excessive dosage is potentially counterproductive, as it decreases iron absorption and increases side effects without improving iron levels or anemia. 23 More research is needed to define the best strategy for oral iron administration.

## When and how to prescribe IV iron? What are the main indications and contraindications?

Intravenous formulations have gained more space in prescriptions as the safety of their use has become more evident. Infusion reactions are rare, usually mild, and if they occur, drug administration can continue at a slower infusion rate. The various injectable formulations have the same efficacy and are especially useful in cases of more vigorous replacement, patients intolerant to oral administration or with malabsorptive processes (for example: patients with inflammatory bowel diseases) and chronic renal patients. Contraindications to use of IV iron are: anemia unrelated to ID, transferrin saturation > 45%, ferritin > 500 ng/mL, active infection/septicemia, severe dysfunction (hepatic or cardiac), pregnant women in the first trimester of pregnancy. Chart 5 shows the IV iron formulations available in Brazil and the main information for the use of these drugs.

**Chart 5.** Intravenous iron formulations

Compound	Ferric hydroxide saccharate	Ferric derisomaltose	Ferric carboxymaltose
Commercial name	Noripurum; Sucrofer	Monofer	Ferinject
Concentration	20 mg/mL	100 mg/mL	50 mg/mL
Total Dose	Determined individually, according to iron deficiency.*	Determined individually, according to iron deficiency.*	Hb < 10 g/dL: 1.500 mg if weight 35-70 kg; 2.000 mg if weight > 70 kg.
	Simplified mean dosage: 100 to 200 mg one to three times a week	Simplified mean dosage: Hb < 10 g/dL: 1.500 mg if weight 35-70 kg; 2.000 mg if weight > 70 kg Hb > 10 g/dL: 1.000 mg if weight 35-70 kg; 1.500 mg if weight > 70 kg.	Hb > 10 g/dL: 1.000 mg if weight 35-70 kg; 1.500 mg if weight > 70 kg.
Recommended maximum single dose	200 mg per day	500 mg daily, up to three times a week	1.000 mg daily, up to once a week
Infusion time	At least 1 hour	15 minutes	15 minutes
Risk category in pregnancy	B	B	B

* **calculation of iron need:** total iron deficiency (mg) = [weight (kg) x DHb (g/dl) x 2.4] + iron reserves (mg)

## Adverse effects of IV iron

Many physicians are reluctant to use IV iron because of concerns about anaphylaxis. True allergic reactions are extremely rare and overrated. In individuals with asthma, inflammatory rheumatic diseases, or multiple drug allergies, premedication with a glucocorticoid alone is generally recommended.

## Final considerations

Although AUB is a very common condition, it should be valued and properly investigated, as it can significantly worsen a woman’s quality of life. According to the etiology, AUB can be effectively treated by quite effective pharmacological and surgical measures depending on age, reproductive desire and other associated conditions. Abnormal uterine bleeding in menacme is the main cause of IDA and ID. The tests requested for diagnosis must include, at least, the blood count, ferritin and iron profile. Iron replacement should be prescribed for these patients, and treatment monitoring is usually performed between 30 and 60 days, depending on the clinical picture.

National Commission Specialized in Venous Thromboembolism and Hemorrhage in Women of the Brazilian Federation of Gynecology and Obstetrics Associations (Febrasgo)

President:

Venina Isabel Poco Viana Leme de Barros

Vice-president:

André Luiz Malavasi Longo de Oliveira

Secretary:

Paulo Francisco Ramos Margarido

Members:

Ana Maria Kondo Igai

Cristiano Caetano Salazar

Dênis José Nascimento

Eduardo Zlotnik

Egle Cristina Couto

Eliane Azeka Hase

Fernanda Andrade Orsi

Joaquim Luiz de Castro Moreira

Marcelo Melzer Teruchkin

Marcos Arêas Marques

Mônica Cristina da Costa Drago Souza

Valeria Doria Mendes da Costa
